# Direction and magnitude of mother–father discrepancies in ADHD symptom ratings: associated factors in a child and adolescent mental health sample

**DOI:** 10.3389/fpsyt.2026.1918797

**Published:** 2026-08-12

**Authors:** YongHao Yang, FangFei Xie, YaoLin Li, JiaQi Song, HuaXia Zhang, YuanYuan Li

**Affiliations:** Mental Health Center, West China Hospital, Sichuan University, Chengdu, China

**Keywords:** ADHD, child and adolescent mental health, clinical assessment, mother–father discrepancies, multi-informant assessment, parental appraisal, parenting consensus, SNAP-IV

## Abstract

**Background/objectives:**

This study examined mother–father discrepancies in ratings of inattention (IA), hyperactivity/impulsivity (HI), and oppositional defiant (OD) symptoms in children and adolescents with ADHD, and explored child, parental, and family factors associated with discrepancy direction and magnitude.

**Methods:**

This single-center cross-sectional clinical study included 460 Chinese children and adolescents aged 6–17 years with clinically confirmed ADHD. Mothers and fathers independently completed the SNAP-IV. Inter-parental agreement was assessed using intraclass correlation coefficients (ICCs), and paired-samples t tests were used to compare maternal and paternal ratings. Standardized signed discrepancy scores were used to examine factors associated with discrepancy direction. Sensitivity analyses were conducted using raw signed discrepancy scores and absolute raw discrepancy scores to examine robustness and disagreement magnitude.

**Results:**

Mothers rated symptom severity significantly higher than fathers across IA, HI, and OD symptoms. Inter-parental agreement was lowest for IA and higher for HI and OD symptoms. Paternal belief that the child had ADHD was consistently associated with discrepancy direction across all three domains. Additional factors associated with discrepancy direction included maternal belief that the child had ADHD and perceived parenting consensus, although their patterns differed across symptom domains. The primary standardized signed discrepancy models explained modest proportions of the variance (R² = 0.055–0.107). In sensitivity analyses, associations between mean standardized symptom level and absolute raw discrepancy were positive and statistically significant in all three domains; corresponding associations with raw signed discrepancy were not statistically significant in any domain.

**Conclusions:**

Mother–father discrepancies in ADHD-related ratings were common and differed across symptom domains. These discrepancies were associated with parental beliefs, perceived parenting consensus, and symptom level. They may offer potentially informative contextual information for multi-informant assessment; however, given the cross-sectional design and modest explanatory power of the regression models, their clinical significance remains to be established.

## Introduction

Attention-deficit/hyperactivity disorder (ADHD) is one of the most common neurodevelopmental disorders in childhood (1, 2). It is characterized by developmentally inappropriate and impairing symptoms of inattention, hyperactivity–impulsivity, or both (1). Recent evidence indicates that ADHD remains common among children and adolescents worldwide and continues to pose a substantial public health burden (3). In China, a recent national survey estimated the prevalence of ADHD at 6.4% among school-attending children and adolescents aged 6–16 years (4). ADHD often persists into adolescence and adulthood and is associated with substantial impairment in academic, family, and social functioning (2, 5). Accurate assessment and early identification are therefore important in child and adolescent mental health services.

The clinical presentation and functional consequences of ADHD vary across children and developmental stages. Inattention may be reflected in difficulties sustaining attention, organizing activities, completing tasks, and following classroom or homework routines, whereas hyperactivity–impulsivity may involve excessive activity, difficulty remaining seated, interrupting, and acting without sufficient forethought. These symptoms can interfere with classroom engagement, homework completion, academic achievement, peer relationships, and family functioning. ADHD-related difficulties may persist into adolescence and adulthood and are associated with substantial educational, social, and family burden (2, 5). Accurate assessment of both symptoms and functional impairment is therefore important for early identification and clinical care.

ADHD assessment commonly relies on information from multiple informants, including parents and teachers, because symptoms may vary across settings and may not be equally visible in all situations (6, 7). As a result, ratings from different informants do not always agree. In the broader child psychopathology literature, such disagreement has been consistently documented and may reflect meaningful differences in context, perspective, and evaluative standards rather than random measurement error alone (8). In ADHD, mothers often report higher symptom severity than fathers, and parent–parent discrepancies may vary across symptom domains (9, 10). Examining these discrepancies may therefore improve understanding of how child symptoms are perceived, interpreted, and communicated within the family context.

Previous studies suggest that parent-report discrepancies in ADHD are not uniform across symptom domains. Disagreement appears to be greater for less directly observable symptoms, such as inattention, and smaller for more overt and behaviorally salient symptoms, such as hyperactivity and impulsivity (10). Symptom-specific work has also shown that parental agreement varies across different types of ADHD-related behaviors rather than following a single global pattern (11). More broadly, discrepancy patterns may be shaped by child characteristics, parent-specific factors, and family-related factors (12, 13). However, less is known about whether factors associated with mother–father discrepancies differ systematically across inattention, hyperactivity/impulsivity, and oppositional defiant symptoms. This question may be especially important because these domains differ in observability, contextual expression, and potential links to family interaction (14, 15). A domain-specific approach may therefore help clarify whether the factors associated with parental disagreement differ across ADHD-related symptom dimensions.

Oppositional defiant (OD) symptoms comprise angry or irritable mood, argumentative or defiant behavior, and vindictiveness. Although OD symptoms and conduct-related problems both belong to the broader externalizing spectrum and may involve interpersonal conflict and noncompliance, they are conceptually distinct. Conduct disorder involves more severe violations of the rights of others or major age-appropriate rules, including aggression, property destruction, deceit or theft, and serious rule violations (15). Accordingly, the present study examined dimensional OD symptoms rather than diagnosing oppositional defiant disorder or assessing the broader range of conduct problems.

In child and adolescent mental health settings, parent-reported symptom ratings are central to ADHD assessment, clinical formulation, and treatment planning. However, mother–father discrepancies may complicate the interpretation of symptom severity, impairment, and family needs. Clarifying the child, parental, and family factors associated with these discrepancies may help clinicians interpret discrepant reports more cautiously and support family-centered assessment and communication in children and adolescents with ADHD.

The present study examined mother–father discrepancies in SNAP-IV ratings in a sample of 460 Chinese children and adolescents with clinically confirmed ADHD. We focused on three symptom domains: inattention (IA), hyperactivity/impulsivity (HI), and oppositional defiant (OD) symptoms. First, we evaluated inter-parental agreement and mean differences in maternal and paternal ratings across these domains. We then examined factors associated with discrepancy direction using standardized signed discrepancy scores, where positive values indicated relatively higher maternal ratings and negative values indicated relatively higher paternal ratings. Finally, we conducted sensitivity analyses using raw signed discrepancy scores and absolute raw discrepancy scores to assess robustness and examine disagreement magnitude. This analytic approach allowed us to distinguish factors associated with which parent reported higher symptom severity from factors associated with the overall magnitude of mother–father disagreement, with implications for ADHD assessment in child and adolescent mental health settings.

## Materials and methods

### Sample

This study was a cross-sectional analysis of baseline data collected as part of an ongoing clinical research program. Participants were consecutively recruited from July 2021 to October 2025, from the outpatient department of the Mental Health Center of a large tertiary hospital in Southwest China. ADHD diagnoses were established through a comprehensive clinical assessment that included a DSM-5-based semi-structured clinical interview conducted according to a fixed clinical interview guide, interviews with the child and caregiver(s), developmental and psychiatric history, mental status examination, and assessment of symptom-related functional impairment (1). The interview covered ADHD symptoms, age at onset, symptom duration, manifestations across settings, functional impairment, and major psychiatric differential diagnoses and comorbidities. The same assessment procedure was used to evaluate the psychiatric exclusion criteria. ADHD diagnoses and psychiatric exclusions were independently confirmed by at least two attending-level or senior clinicians, each with more than 10 years of clinical experience.

Information regarding symptoms and impairment outside the home was incorporated into the diagnostic assessment to confirm the cross-situational manifestations required by DSM-5. Depending on the information available for each participant, school-related information was obtained either directly from teachers or indirectly from caregivers based on feedback they had received from teachers regarding the child’s classroom behavior, attention, academic functioning, and overall school adjustment. Direct teacher-reported information was therefore not available for every participant. The maternal and paternal SNAP-IV ratings analyzed in the present study were not used to establish the ADHD diagnosis or determine study eligibility, and no SNAP-IV cutoff score or mother–father agreement or discrepancy criterion was applied during participant selection. The inclusion criteria were: (1) age 6–17 years; (2) gestational age ≥34 weeks and birth weight ≥2,500 g, to reduce potential confounding effects of prematurity or low birth weight on neurodevelopment; and (3) meeting DSM-5 diagnostic criteria for ADHD. Exclusion criteria were: (1) co-occurring autism spectrum disorder, schizophrenia, bipolar disorder, anxiety disorders, depressive disorders, Tourette syndrome, or other major psychiatric disorders; (2) severe physical illness, such as refractory epilepsy, congenital heart disease, or organic brain disease; (3) diagnosed intellectual disability or full-scale IQ <70; (4) use of psychostimulants or other psychiatric medications within the preceding month, or concurrent participation in another ADHD intervention study; and (5) severe mental illness or cognitive impairment in one or both parents that could compromise rating validity.

Because recruitment was embedded in routine outpatient care, the total number of patients assessed before enrolment and the number declining participation could not be reliably retrieved. A total of 467 clinically eligible children and adolescents were enrolled. Seven records were excluded during data cleaning for reasons unrelated to the availability of maternal or paternal SNAP-IV ratings. No participant was excluded because maternal or paternal SNAP-IV ratings were unavailable. The final analytic sample comprised 460 children and adolescents aged 6–17 years (M = 9.02, SD = 2.26), of whom 80.4% were male. The study protocol was approved by the Biomedical Research Ethics Committee of West China Hospital, Sichuan University, and written informed consent was obtained from the parents or legal guardians of all participants before data collection.

### SNAP-IV rating scale

The SNAP-IV consists of 26 items, including 18 core ADHD symptoms, with 9 items assessing inattention and 9 items assessing hyperactivity/impulsivity, as well as 8 items assessing oppositional defiant (OD) symptoms. The OD symptom items cover angry or irritable mood, argumentative or defiant behavior, and vindictiveness. In the present study, the subscale was treated as a dimensional measure of OD symptoms and was not used to establish a diagnosis of oppositional defiant disorder or to assess conduct disorder. Mothers and fathers completed the SNAP-IV and parent-specific questionnaire items independently and were not permitted to view each other’s responses during completion. Each parent rated the severity of the SNAP-IV items over the past 4 weeks on a 4-point Likert scale ranging from 0 (not at all) to 3 (very much). Questionnaire respondents were identified as the child’s mother and father. However, biological-parent status, co-residence with the child, and primary-caregiver status were not systematically recorded. Subscale total scores were calculated for the inattention, hyperactivity/impulsivity, and oppositional defiant symptom domains to compare maternal and paternal ratings. The Chinese version of the SNAP-IV parent form has demonstrated good psychometric properties (16) and has been used in recent Chinese ADHD-related studies (17, 18). In the present study, Cronbach’s alpha coefficients indicated high internal consistency across all subscales. For mother-reported ratings, Cronbach’s alpha was 0.88 for inattention, 0.86 for hyperactivity/impulsivity, and 0.88 for oppositional defiant symptoms. For father-reported ratings, Cronbach’s alpha was 0.89 for inattention, 0.87 for hyperactivity/impulsivity, and 0.89 for oppositional defiant symptoms. Although ADHD diagnoses were based on the comprehensive clinical assessment described above rather than on the SNAP-IV alone, the SNAP-IV was the only standardized symptom-rating instrument included in the present analyses. It was selected because the study was specifically designed to compare maternal and paternal ratings obtained using the same items and response scale across the prespecified symptom domains. Complementary standardized teacher-report, child self-report, behavioral, and functional-impairment measures were not included in the baseline protocol.

### Assessment of child and family background variables

To assess potential factors associated with mother–father rating discrepancies, we developed a Comprehensive Questionnaire on Child and Family Background for the present study. The questionnaire was informed by prior research on informant discrepancies and parent-related factors in child psychopathology (8, 11, 12, 19–21). It covered three domains: (1) demographic and clinical characteristics, including family structure, perinatal factors, sleep problems, and parental education; (2) parental health-related characteristics, including parental physical and mental illness history and childhood ADHD-like symptoms; and (3) parental appraisal and family-context variables, including parental belief that the child had ADHD, perceived parent–child relationship quality, and perceived parenting consensus. Parental belief that the child had ADHD was assessed separately for mothers and fathers using single items completed during the baseline clinical assessment and before diagnostic feedback was provided to the family. Each parent was asked whether they believed that the child had ADHD. Responses were coded from 1 to 3: 1 = definitely not, 2 = possibly, and 3 = definitely, with higher scores indicating a stronger belief that the child had ADHD. Perceived parenting consensus was assessed separately for mothers and fathers using single items asking whether each parent perceived that they could reach consensus with the other parent in child-rearing. Responses were coded from 1 to 4, with higher scores indicating greater perceived parenting consensus: 1 = not at all, 2 = not very much, 3 = mostly, and 4 = completely.

### Data analysis

All statistical analyses were conducted using SPSS 26.0, and figures were generated in R 4.5.3. Maternal and paternal mean ratings on the SNAP-IV subscales were first compared using paired-samples t tests. Inter-parental agreement was then evaluated by calculating intraclass correlation coefficients (ICCs) for the SNAP-IV inattention (IA), hyperactivity/impulsivity (HI), and OD symptom subscale, as well as for each individual SNAP-IV item. Because mothers and fathers were treated as two fixed informants for each child, two-way mixed-effects, absolute-agreement, single-measure ICCs, was used. ICC values were interpreted with reference to Shrout (22) and Shrout and Fleiss (23).

Hierarchical multiple linear regression analyses were performed to examine factors associated with mother–father discrepancies. For each symptom domain, maternal and paternal raw subscale total scores were standardized separately within the maternal and paternal rating distributions. The primary dependent variable, termed the standardized signed discrepancy, was calculated as the maternal z-score minus the paternal z-score, following De Los Reyes and Kazdin (24) and Langberg et al. (11). This score represents the difference between the child’s relative standing in the maternal rating distribution and the child’s relative standing in the paternal rating distribution. Positive values indicate a relatively higher position in the maternal rating distribution, whereas negative values indicate a relatively higher position in the paternal rating distribution. Because maternal and paternal scores were standardized separately, the standardized signed discrepancy is not numerically equivalent to the raw maternal-minus-paternal difference and should not be interpreted as the number of raw SNAP-IV points separating the two ratings. For sensitivity analyses, raw signed discrepancy scores were calculated as maternal raw scores minus paternal raw scores, such that positive values indicated higher maternal raw ratings and negative values indicated higher paternal raw ratings. To examine discrepancy magnitude, absolute raw discrepancy scores were calculated as |maternal raw score − paternal raw score|. For the regression analyses, mean standardized symptom level for each domain was calculated as (maternal z-score + paternal z-score)/2. For descriptive visualizations, mean raw parental ratings were calculated as (maternal raw score + paternal raw score)/2.

Following previous ADHD parent-discrepancy research (10), candidate associated factors were first screened through bivariate analyses and were entered into the corresponding domain-specific regression model if they showed preliminary associations at p < 0.20. This liberal screening threshold was used to reduce the risk of excluding potentially relevant variables at the preliminary selection stage. In Step 1, demographic, clinical, and family-background variables were entered, including mean standardized symptom level for the corresponding domain. In Step 2, parental appraisal and family-context variables, including maternal and paternal belief that the child had ADHD and perceived parenting consensus, were entered. Binary variables were dummy-coded, and higher scores on ordinal indicators reflected greater levels, better states, or more positive attitudes, as applicable. Variance inflation factors (VIFs) were examined to assess multicollinearity, and all VIFs were below 2.0. Bland–Altman plots were used to display signed mother–father differences, whereas scatterplots were used to illustrate the association between mean parental ratings and absolute raw mother–father discrepancies across IA, HI, and OD symptoms (25). Cases with missing values in variables included in each regression model were handled using listwise deletion in SPSS.

## Results

### Mother–father differences and inter-parental agreement in SNAP-IV ratings

Before examining factors associated with mother–father discrepancies, we first assessed whether systematic differences existed between maternal and paternal ratings. Paired-samples t tests showed that maternal ratings were significantly higher than paternal ratings across all three symptom domains (see **Table 1**). Intraclass correlation coefficients (ICCs) were then calculated to assess inter-parental agreement on the 26 items and subscale total scores of the SNAP-IV. As shown in **Table 2**, ICC values for individual items ranged from 0.153 to 0.496. Item 24 (“Touchy or easily annoyed”) demonstrated the highest agreement (ICC = 0.496), whereas Item 8 (“Easily distracted”) showed the lowest agreement (ICC = 0.153). At the domain level, agreement was higher for HI (ICC = 0.474) and OD symptoms (ICC = 0.469) than for IA (ICC = 0.331). The ICC for the 18-item ADHD core symptom total was 0.374. Overall, agreement was generally higher for more overt externalizing symptoms, such as hyperactivity, impulsivity, and oppositional behaviors, than for inattentive symptoms.

**Table 1:** Comparison of maternal and paternal SNAP-IV inattention, hyperactivity/impulsivity, and oppositional defiant symptom scores.

Rating	*N*	Mothers, M (SD)	Fathers, M (SD)	Paired difference (95% CI)	*t*	*p*	Cohen’s *d_z_*
IA	460	15.32 (5.52)	13.86 (5.31)	1.46 (0.89, 2.03)	5.052	< 0.001	0.236
HI	460	10.08 (5.36)	9.23 (5.22)	0.85 (0.36, 1.35)	3.387	< 0.001	0.158
OD symptoms	460	9.41 (4.63)	8.20 (4.53)	1.21 (0.79, 1.64)	5.599	< 0.001	0.261

Paired differences were calculated as maternal ratings minus paternal ratings; positive values indicate higher maternal ratings. IA, inattention; HI, hyperactivity/impulsivity; OD symptoms, oppositional defiant symptoms; CI, confidence interval; SD, standard deviation. Cohen’s dz was calculated for paired-samples comparisons as t/√n.

**Table 2:** Mother–father agreement on SNAP-IV inattention, hyperactivity/impulsivity, and oppositional defiant symptom ratings.

Item no.	ADHD symptom description (abbr.)	Mother × father ICC	95% CI
1	Makes careless mistakes	0.219	(0.131, 0.304)
2	Difficulty sustaining attention	0.203	(0.115, 0.288)
3	Does not listen when spoken to	0.248	(0.160, 0.331)
4	Fails to finish tasks	0.302	(0.217, 0.383)
5	Difficulty organizing tasks	0.210	(0.121, 0.295)
6	Avoids sustained mental effort	0.264	(0.178, 0.347)
7	Loses things	0.381	(0.300, 0.456)
8	Easily distracted	0.153	(0.064, 0.239)
9	Forgetful in daily activities	0.307	(0.220, 0.389)
10	Fidgets with hands or feet	0.270	(0.183, 0.354)
11	Leaves seat	0.443	(0.367, 0.514)
12	Runs about or climbs excessively	0.483	(0.410, 0.550)
13	Difficulty playing quietly	0.328	(0.244, 0.407)
14	On the go/Driven by a motor	0.366	(0.285, 0.443)
15	Talks excessively	0.463	(0.379, 0.538)
16	Blurts out answers	0.281	(0.192, 0.364)
17	Difficulty awaiting turn	0.341	(0.258, 0.419)
18	Interrupts or intrudes on others	0.288	(0.200, 0.371)
19	Loses temper	0.443	(0.361, 0.517)
20	Argues with adults	0.310	(0.222, 0.392)
21	Defies or refuses requests	0.311	(0.225, 0.392)
22	Deliberately annoys people	0.354	(0.272, 0.431)
23	Blames others for mistakes	0.282	(0.196, 0.363)
24	Touchy or easily annoyed	0.496	(0.390, 0.584)
25	Angry and resentful	0.372	(0.291, 0.448)
26	Spiteful or vindictive	0.368	(0.286, 0.444)
Total	Inattention (IA) Total Score	0.331	(0.243, 0.413)
Total	Hyperactivity/Impulsivity (HI) Total Score	0.474	(0.398, 0.542)
Total	Oppositional Defiant (OD) Symptom Total Score	0.469	(0.383, 0.545)
Total	ADHD Core Symptoms (18 items) Total Score	0.374	(0.289, 0.453)

ICC, Intraclass Correlation Coefficient; CI, Confidence Interval. Items are based on the SNAP-IV rating scale.

**Figure 1:**
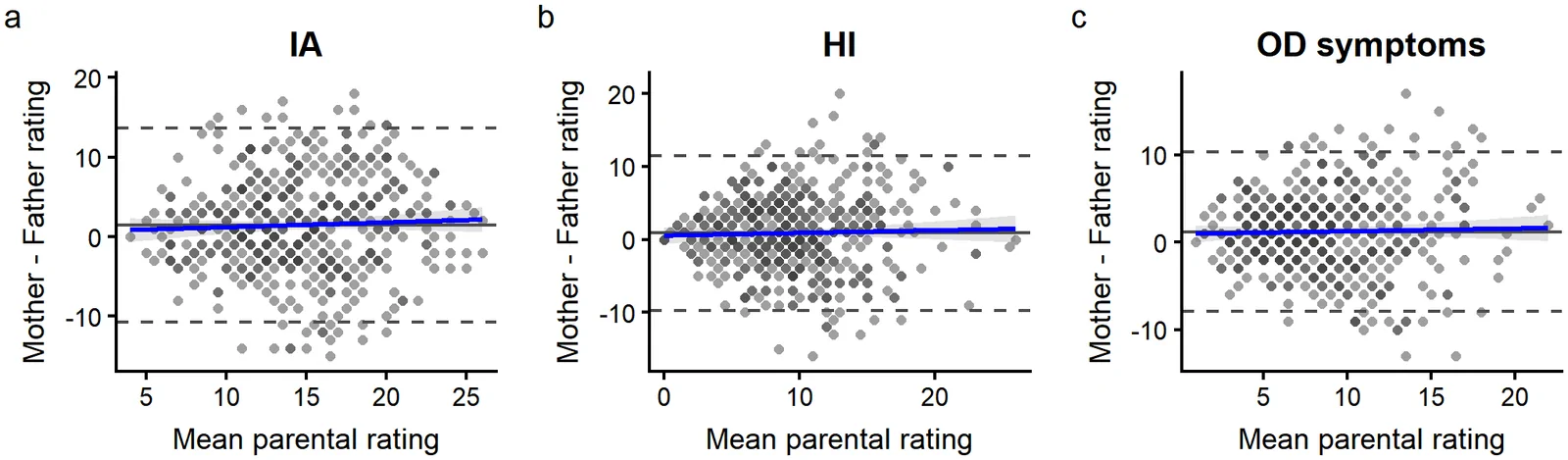
Bland–Altman plots of mother–father differences in raw ratings of IA, HI, and oppositional defiant symptoms. Panels represent **(a)** IA, **(b)** HI, and **(c)** OD symptoms. The x-axis represents the mean raw parental rating, calculated as (maternal raw score + paternal raw score)/2, and the y-axis represents the raw mother–father difference (maternal raw score − paternal raw score). The solid horizontal line indicates the mean difference, and the dashed lines indicate the 95% limits of agreement. The blue fitted line and shaded area indicate the fitted linear trend and its 95% confidence interval.

Bland–Altman plots were constructed for the IA, HI, and OD symptom domains to further illustrate patterns of inter-parental disagreement (**Figure 1**). Across all three panels, the mean mother–father difference was above zero, indicating that maternal ratings were generally higher than paternal ratings. In addition, visual inspection of the fitted trend lines suggested no clear proportional pattern in signed discrepancies across symptom levels.

### Factors associated with standardized signed discrepancies

#### Inattention domain

The final regression model for the IA domain was statistically significant, F(13, 446) = 3.273, p < 0.001, explaining 8.7% of the variance in standardized signed mother–father discrepancy scores (R² = 0.087). As shown in **Table 3**, paternal belief that the child had ADHD was associated with lower standardized signed discrepancy scores (β = −0.194, p < 0.001), whereas maternal belief that the child had ADHD (β = 0.121, p = 0.021) and paternal perceived parenting consensus (β = 0.124, p = 0.010) were associated with higher standardized signed discrepancy scores. Mean standardized IA symptom level was not significantly associated with standardized signed discrepancy scores (β = −0.014, p = 0.774). These findings suggest that the direction of mother–father discrepancy in IA ratings was more closely related to parental ADHD-related appraisals and paternal perceived parenting consensus than to overall IA symptom severity.

**Table 3:** Multiple regression models examining factors associated with standardized signed mother–father discrepancies across IA, HI, and OD symptoms.

Associated factors	IA: B (95% CI); β; p	HI: B (95% CI); β; p	OD symptoms: B (95% CI); β; p
Demographic and clinical factors			
Mean standardized symptom level	−0.020 (−0.155, 0.115); −0.014;.774	0.027 (−0.088, 0.143); 0.023;.641	−0.060 (−0.172, 0.051); −0.051;.286
Body weight	−0.005 (−0.014, 0.003); −0.059;.205	—	—
Foster care history	0.257 (−0.017, 0.531); 0.087;.066	—	0.286 (0.049, 0.522); 0.109;.018
Delivery mode	−0.061 (−0.276, 0.155); −0.026;.580	—	—
Paternal childhood ADHD-like symptoms	−0.225 (−0.474, 0.025); −0.082;.077	−0.196 (−0.422, 0.030); −0.080;.090	—
Maternal childhood ADHD-like symptoms	—	−0.168 (−0.432, 0.096); −0.059;.211	—
Sleep problems	0.116 (−0.098, 0.330); 0.050;.287	—	0.180 (−0.010, 0.371); 0.087;.063
Biological family status	0.164 (−0.259, 0.586); 0.035;.446	—	—
Paternal education	0.095 (−0.092, 0.281); 0.046;.318	0.062 (−0.133, 0.257); 0.034;.533	—
Maternal familiarity with the child	−0.356 (−0.761, 0.049); −0.081;.085	—	—
Child sex	—	0.170 (−0.069, 0.409); 0.066;.162	0.288 (0.060, 0.515); 0.112;.013
Birth injury	—	−0.148 (−0.540, 0.244); −0.035;.458	—
Maternal education	—	0.097 (−0.110, 0.304); 0.051;.357	0.081 (−0.090, 0.252); 0.043;.351
Somatic illness after the neonatal period	—	—	0.150 (−0.031, 0.331); 0.074;.104
Full-term birth	—	—	0.243 (−0.064, 0.551); 0.071;.120
Maternal severe physical illness	—	—	0.401 (−0.252, 1.054); 0.055;.228
Parental appraisal and family-context variables			
Maternal belief that the child had ADHD	0.234 (0.036, 0.433); 0.121;.021	0.027 (−0.148, 0.202); 0.016;.763	0.245 (0.077, 0.413); 0.143;.004
Paternal belief that the child had ADHD	−0.352 (−0.533, −0.171); −0.194; <.001	−0.215 (−0.379, −0.050); −0.133;.011	−0.174 (−0.334, −0.014); −0.108;.033
Maternal perceived parenting consensus	−0.133 (−0.281, 0.015); −0.087;.078	−0.147 (−0.277, −0.016); −0.107;.027	−0.201 (−0.330, −0.071); −0.147;.002
Paternal perceived parenting consensus	0.238 (0.057, 0.419); 0.124;.010	0.137 (−0.027, 0.300); 0.080;.101	0.213 (0.054, 0.372); 0.125;.009

B, unstandardized coefficient; β, standardized coefficient; CI, confidence interval. The outcome was maternal z-score − paternal z-score; coefficient signs indicate relatively higher maternal (+) or paternal (−) ratings. Dashes denote variables not included in the domain-specific model. N = 460 for IA and 459 for HI and OD symptoms. See **Supplementary Table 1** for variable coding.

#### Hyperactivity/impulsivity domain

The final regression model for the HI domain was statistically significant, F(11, 447) = 2.369, p = 0.007, explaining 5.5% of the variance in standardized signed mother–father discrepancy scores (R² = 0.055). As presented in **Table 3**, maternal perceived parenting consensus was associated with lower standardized signed discrepancy scores (β = −0.107, p = 0.027), and paternal belief that the child had ADHD was also associated with lower standardized signed discrepancy scores (β = −0.133, p = 0.011). Mean standardized HI symptom level was not significantly associated with standardized signed discrepancy scores (β = 0.023, p = 0.641). These findings indicate that discrepancy direction in HI ratings was associated with paternal belief that the child had ADHD and maternal perceived parenting consensus, rather than with overall HI symptom severity.

#### Oppositional defiant symptom domain

The final regression model for the oppositional defiant symptom domain was statistically significant, F(12, 446) = 4.436, p < 0.001, explaining 10.7% of the variance in standardized signed mother–father discrepancy scores (R² = 0.107). As shown in **Table 3**, maternal belief that the child had ADHD (β = 0.143, p = 0.004), foster care history (β = 0.109, p = 0.018), child sex (β = 0.112, p = 0.013), and paternal perceived parenting consensus (β = 0.125, p = 0.009) were associated with higher standardized signed discrepancy scores. In contrast, paternal belief that the child had ADHD (β = −0.108, p = 0.033) and maternal perceived parenting consensus (β = −0.147, p = 0.002) were associated with lower standardized signed discrepancy scores. Mean standardized oppositional defiant symptom level was not significantly associated with standardized signed discrepancy scores (β = −0.051, p = 0.286). These findings suggest that the direction of mother–father discrepancy in oppositional defiant symptom ratings was associated with parental ADHD-related appraisals, perceived parenting consensus, and selected child and family background factors.

### Sensitivity analyses

**Figure 2:**
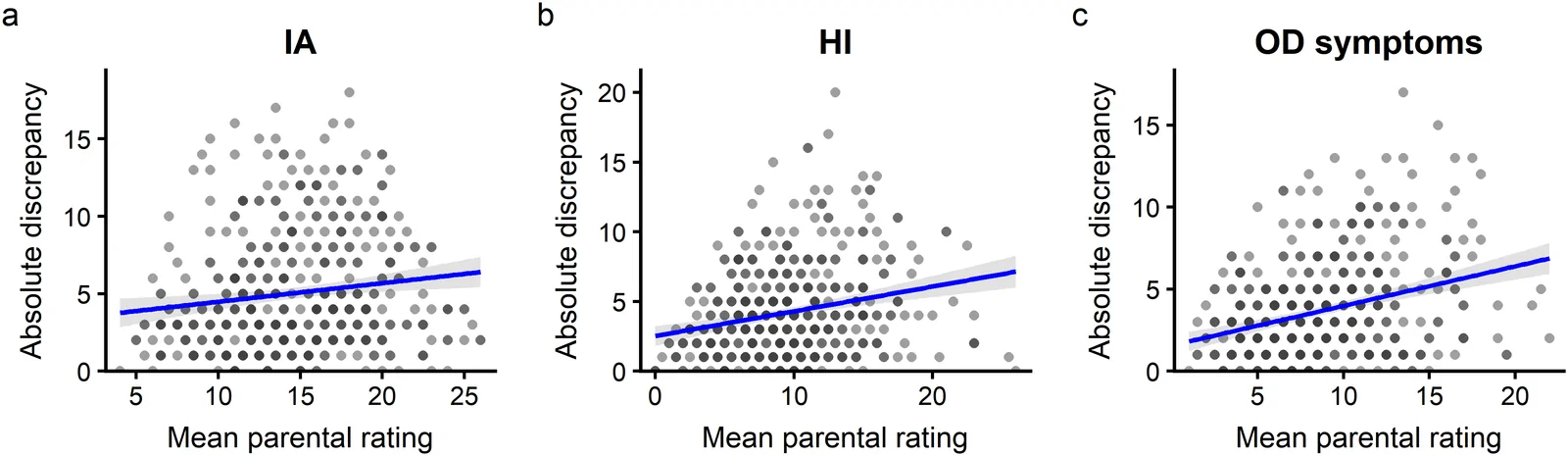
Scatterplots of the association between mean raw parental ratings and absolute raw mother–father discrepancies in IA, HI, and OD symptoms. Panels represent **(a)** IA, **(b)** HI, and **(c)** OD symptoms. The x-axis represents the mean raw parental rating, calculated as (maternal raw score + paternal raw score)/2, and the y-axis represents the absolute raw mother–father discrepancy, defined as |maternal raw score − paternal raw score|. The blue line and shaded area indicate the fitted linear trend and its 95% confidence interval.

Sensitivity analyses using raw signed discrepancy scores, calculated as maternal raw scores minus paternal raw scores, yielded results that were substantively consistent with the primary standardized signed discrepancy models (**Supplementary Table 2**). The set and direction of statistically significant associations were unchanged across all three symptom domains, and minor numerical differences among nonsignificant coefficients did not alter the overall interpretation. When absolute raw discrepancy scores were used as outcome variables, the regression results presented in **Table 4** showed that mean symptom severity was positively associated with discrepancy magnitude in all three symptom domains. In the IA model, greater discrepancy magnitude was associated with higher mean IA severity, foster care history, and higher paternal education, whereas paternal childhood ADHD-like symptoms and maternal perceived parenting consensus were associated with smaller discrepancy magnitude. In the HI model, discrepancy magnitude was associated only with mean HI severity. In the OD symptom model, greater discrepancy magnitude was associated with higher mean OD symptom level, foster care history, and maternal severe physical illness. **Figure 2** presents descriptive scatterplots of mean raw parental ratings and absolute raw mother–father discrepancies. The fitted lines showed positive slopes across all three symptom domains. Because mean symptom level and absolute discrepancy were derived from the same pair of bounded maternal and paternal ratings, these associations should be interpreted cautiously and may partly reflect mathematical coupling.

**Table 4:** Multiple regression models examining factors associated with the magnitude of mother–father discrepancies across IA, HI, and OD symptoms.

Associated factors	IA: B (95% CI); β; p	HI: B (95% CI); β; p	OD symptoms: B (95% CI); β; p
Demographic and clinical factors			
Mean standardized symptom level	0.678 (0.217, 1.138); 0.142;.004	0.964 (0.578, 1.349); 0.241; <.001	1.025 (0.692, 1.357); 0.289; <.001
Body weight	0.006 (−0.023, 0.034); 0.018;.700	—	—
Foster care history	1.149 (0.215, 2.082); 0.114;.016	—	0.724 (0.019, 1.429); 0.092;.044
Delivery mode	0.451 (−0.283, 1.185); 0.056;.228	—	—
Paternal childhood ADHD-like symptoms	−0.885 (−1.736, −0.035); −0.094;.041	0.043 (−0.712, 0.798); 0.005;.911	—
Maternal childhood ADHD-like symptoms	—	−0.664 (−1.545, 0.217); −0.069;.139	—
Sleep problems	0.362 (−0.368, 1.091); 0.045;.331	—	−0.197 (−0.765, 0.371); −0.031;.496
Biological family status	0.821 (−0.618, 2.260); 0.052;.263	—	—
Paternal education	0.740 (0.104, 1.376); 0.106;.023	0.599 (−0.054, 1.251); 0.097;.072	—
Maternal familiarity with the child	−1.082 (−2.462, 0.298); −0.072;.124	—	—
Child sex	—	−0.050 (−0.847, 0.746); −0.006;.901	0.216 (−0.463, 0.895); 0.028;.532
Birth injury	—	0.603 (−0.704, 1.910); 0.042;.365	—
Maternal education	—	−0.054 (−0.745, 0.637); −0.008;.879	0.217 (−0.293, 0.726); 0.038;.403
Somatic illness after the neonatal period	—	—	−0.191 (−0.731, 0.348); −0.031;.486
Full-term birth	—	—	0.653 (−0.264, 1.571); 0.063;.162
Maternal severe physical illness	—	—	2.441 (0.491, 4.390); 0.111;.014
Parental appraisal and family-context variables			
Maternal belief that the child had ADHD	−0.531 (−1.208, 0.145); −0.080;.123	0.170 (−0.415, 0.756); 0.029;.567	0.002 (−0.500, 0.504); 0.000;.993
Paternal belief that the child had ADHD	−0.430 (−1.048, 0.187); −0.069;.171	−0.300 (−0.848, 0.248); −0.055;.283	−0.096 (−0.574, 0.381); −0.020;.692
Maternal perceived parenting consensus	−0.518 (−1.023, −0.013); −0.099;.044	−0.237 (−0.672, 0.197); −0.051;.284	−0.206 (−0.591, 0.180); −0.050;.295
Paternal perceived parenting consensus	0.328 (−0.290, 0.945); 0.050;.297	0.154 (−0.391, 0.699); 0.027;.579	−0.172 (−0.646, 0.301); −0.034;.475

B, unstandardized coefficient; β, standardized coefficient; CI, confidence interval. The outcome was |maternal raw score − paternal raw score|; coefficient signs indicate greater (+) or smaller (−) discrepancy. Dashes denote variables not included in the domain-specific model. N = 460 for IA and 459 for HI and OD symptoms. See **Supplementary Table 1** for variable coding.

## Discussion

This study examined mother–father agreement and discrepancies in SNAP-IV ratings among Chinese children and adolescents with clinically confirmed ADHD. The findings suggest that mother–father discrepancies in ADHD-related symptom ratings should not be interpreted simply as random measurement error, but may offer potentially informative contextual information about parental appraisal, perceived parenting consensus, family context, and symptom severity. Three main findings emerged. First, mothers rated IA, HI, and OD symptoms as more severe than fathers, and inter-parental agreement varied across symptom domains, with the weakest agreement observed for IA. This pattern is consistent with previous multi-informant studies showing that ADHD symptom ratings may differ across informants and symptom domains (8, 9, 13, 26, 27). Second, factors associated with discrepancy direction were primarily related to parental beliefs that the child had ADHD and perceived parenting consensus, supporting the view that parent-specific appraisal and family context may shape which parent reports higher symptom severity (28). Third, sensitivity analyses showed that symptom severity was more consistently associated with discrepancy magnitude than with discrepancy direction, suggesting that signed and absolute discrepancy scores capture different aspects of mother–father disagreement. Overall, these findings support the distinction between discrepancy direction and discrepancy magnitude and highlight the potential value of discrepant parental reports for ADHD assessment and family-centered clinical communication. The family-context findings in the present study should be interpreted within the broader literature on ADHD symptoms, parenting practices, and family functioning. A Turkish case–control study reported differences in parenting practices and overall family functioning between families of children with ADHD and control families; within one ADHD presentation subgroup, poorer family functioning was also associated with greater ADHD symptom severity (29). Similarly, an Australian three-year longitudinal study identified persistent difficulties across multiple family domains among families of children with ADHD or subthreshold ADHD, including parenting consistency, parental wellbeing, parent–partner support, parenting warmth, and family quality of life (30). These studies indicate that ADHD symptom burden may coexist with difficulties across several levels of family functioning rather than being related to a single parenting characteristic. In the present study, perceived parenting consensus may reflect parents’ coordination in child-rearing, while factors such as foster care history and maternal severe physical illness may indicate broader differences in caregiving circumstances. The domain-specific and partly inconsistent associations observed in the present study therefore suggest that mother–father rating discrepancies may occur within heterogeneous family contexts, rather than reflecting one uniform family mechanism.

### Factors associated with standardized signed discrepancies and absolute discrepancy magnitude in IA ratings

IA showed the lowest level of mother–father agreement among the three symptom domains, which is consistent with the less observable and more context-dependent nature of inattentive symptoms. Unlike overt hyperactive or oppositional behaviors, inattention may become most apparent during homework, sustained task engagement, classroom routines, and daily organization. Mothers and fathers may not observe these contexts to the same extent, which may increase ambiguity in parental judgments and contribute to lower inter-parental agreement (31). In this context, parental beliefs that the child had ADHD and perceived parenting consensus may influence how inattentive behaviors are noticed, interpreted, and translated into symptom ratings (21, 32). The sensitivity analyses suggested a different pattern for discrepancy magnitude. Mean IA severity was associated with the absolute size of mother–father disagreement, but not with discrepancy direction. Foster care history and higher paternal education were associated with greater discrepancy magnitude, whereas paternal childhood ADHD-like symptoms and maternal perceived parenting consensus were associated with smaller discrepancy magnitude. These findings suggest that parental appraisal may be more relevant to discrepancy direction, whereas symptom severity and selected family-context factors may be more relevant to the overall distance between mother and father ratings.

### Factors associated with standardized signed discrepancies and absolute discrepancy magnitude in HI ratings

Mother–father agreement was higher for HI than for IA, which may reflect the more overt and behaviorally salient nature of hyperactive and impulsive symptoms (11, 14). Behaviors such as leaving one’s seat, interrupting others, excessive talking, and motor restlessness are generally easier to observe across everyday settings, which may reduce ambiguity about whether the behavior occurred. However, greater observability does not eliminate parent-specific interpretation. Parents may still differ in how they judge the severity, acceptability, or impairment of the same behavior (31).

In the present study, discrepancy direction in HI ratings was associated with paternal belief that the child had ADHD and maternal perceived parenting consensus. This suggests that parental appraisal may still influence which parent rates HI symptoms as more severe, even in a relatively visible symptom domain. By contrast, the sensitivity analyses showed that mean HI severity was associated with discrepancy magnitude, whereas other factors in the HI magnitude model were not statistically significant. One possible explanation is that more severe HI symptoms are more frequent and salient in daily life, increasing the overall distance between mother and father ratings without necessarily determining which parent reports higher symptom severity. Thus, HI discrepancies may reflect both symptom visibility and parent-specific interpretation, with symptom severity more relevant to discrepancy magnitude and parental appraisal more relevant to discrepancy direction.

### Factors associated with standardized signed discrepancies and absolute discrepancy magnitude in OD symptom ratings

Among the three symptom domains, the OD symptom model explained the largest proportion of variance in discrepancy direction, although the explained variance remained modest. This pattern suggests that mother–father discrepancies in OD symptom ratings may be especially sensitive to parental appraisal and family-context factors. Unlike inattentive or hyperactive/impulsive symptoms, oppositional and defiant behaviors are often expressed in relational contexts involving discipline, compliance, authority, and conflict (15). Therefore, OD symptom ratings may depend not only on what parents observe, but also on how they interpret the child’s behavior within parent–child and broader family interactions. In the present study, parental beliefs that the child had ADHD and perceived parenting consensus were associated with discrepancy direction in OD symptom ratings. The opposite associations observed for maternal and paternal perceived parenting consensus should be interpreted cautiously. Maternal perceived parenting consensus was associated with lower signed discrepancy scores, whereas paternal perceived parenting consensus was associated with higher signed discrepancy scores. This may indicate that mothers and fathers evaluate parenting agreement from different positions within the family, or that perceived consensus does not represent the same relational experience for both parents. These findings are consistent with the view that OD symptom discrepancies may reflect not only child behavior itself, but also the family and relational contexts in which oppositional behavior is interpreted (12, 33–35). The sensitivity analyses further showed that mean OD symptom level, foster care history, and maternal severe physical illness were associated with greater discrepancy magnitude. This suggests that mother–father disagreement may become larger when oppositional symptoms are more severe or when family circumstances are more complex. In such cases, both parents may recognize behavioral difficulties, but differ more widely in the degree of concern, burden, or impairment they attribute to those behaviors.

### Implications of distinguishing discrepancy direction from discrepancy magnitude

In the present study, mean symptom severity was not associated with discrepancy direction in IA, HI, or OD symptoms, but was consistently associated with discrepancy magnitude in the sensitivity analyses. In contrast, parental beliefs that the child had ADHD and perceived parenting consensus were more consistently associated with discrepancy direction. This pattern suggests that discrepancy direction and discrepancy magnitude capture different aspects of mother–father disagreement. Symptom severity may increase the salience and burden of child behaviors in daily life, thereby widening the overall distance between maternal and paternal ratings. However, symptom severity did not determine which parent rated symptoms as more severe. By contrast, parental beliefs that the child had ADHD and perceived parenting consensus may reflect parent-specific evaluative positions and may therefore be more relevant to discrepancy direction. These findings suggest that signed and absolute discrepancy scores should not be treated as interchangeable indicators. Signed scores indicate which parent reports relatively higher symptom severity, whereas absolute scores indicate how far apart the two parents’ ratings are. Distinguishing these two forms of discrepancy may help clarify whether parental disagreement is more closely related to parent-specific appraisal or to overall symptom burden. This interpretation is consistent with multi-informant assessment frameworks suggesting that informant discrepancies may reflect meaningful differences in context, perspective, and assessment purpose, rather than measurement error alone (27, 36).

### Clinical implications for child and adolescent mental health assessment

These findings are clinically relevant for child and adolescent mental health assessment because informant discrepancies may influence how clinicians evaluate symptom severity, functional impairment, service needs, and treatment goals (37, 38). Mother–father disagreement should not automatically be regarded as random measurement error or invalid reporting. Instead, discrepant parental ratings may provide useful information about parental appraisals, observation contexts, family-context factors, and symptom burden. Clinicians should therefore consider the symptom domain, the direction of the discrepancy, and the magnitude of disagreement when integrating maternal and paternal reports. In practice, discrepancies in IA ratings may prompt clinicians to ask which parent more closely observes homework, sustained task engagement, daily organization, and school-related routines. For HI and OD symptoms discrepancies, it may be useful to explore impairment thresholds, discipline contexts, parent–child conflict, and perceived parenting consensus. Greater disagreement in more severe cases should also not be dismissed as poor rating validity. Rather, it may indicate more complex symptom expression and family response, requiring more careful integration of information from both parents. Discussing these differences directly with families may help develop a shared formulation and support more family-centered ADHD assessment and communication.

## Limitations

Several limitations should be acknowledged. First, the cross-sectional design precludes causal inference, and the modest variance explained by the regression models indicates that the identified associations should be regarded as exploratory and that their clinical significance remains to be established. Second, the single-center, predominantly male clinical sample may limit generalizability. Although excluding major psychiatric comorbidities increased sample homogeneity and reduced potential confounding, it may restrict applicability to routine clinical populations, in which ADHD comorbidity is common. Third, the SNAP-IV was the only standardized symptom-rating instrument included in the present analyses, and teacher ratings, developmentally appropriate child or adolescent self-reports, complementary standardized measures, and independent external criteria were unavailable. Consequently, multimethod validation was not possible, and the study cannot determine which parent provided a more accurate rating; future studies should incorporate these additional informants and assessment methods. Fourth, several family-context variables were assessed using single items, while biological-parent status, co-residence with the child, and primary-caregiver status were not systematically recorded. Parental belief that the child had ADHD may also overlap with the same parent’s perception of the child’s symptoms. Finally, mean standardized symptom level and absolute raw discrepancy were derived from the same bounded maternal and paternal ratings. Their association may therefore reflect both substantive differences and mathematical coupling and should be interpreted cautiously.

## Conclusion

In summary, mother–father discrepancies in SNAP-IV ratings among Chinese children and adolescents with ADHD were common and varied across symptom domains. Mothers generally rated IA, HI, and OD symptoms as more severe than fathers, and inter-parental agreement was weakest for IA. Parental beliefs that the child had ADHD and perceived parenting consensus were more consistently associated with discrepancy direction, whereas symptom severity was consistently associated with discrepancy magnitude. These findings suggest that mother–father discrepancies are not merely measurement noise in ADHD assessment, but may offer potentially informative contextual information about parental appraisal, family context, and symptom burden. Distinguishing discrepancy direction from discrepancy magnitude may help clinicians integrate discrepant parental reports more systematically in child and adolescent mental health assessment.

## Data Availability

The raw data supporting the conclusions of this article will be made available by the authors, without undue reservation.
