# Supplementary material for: Direction and magnitude of mother–father discrepancies in ADHD symptom ratings: associated factors in a child and adolescent mental health sample
Source: Front Psychiatry. 2026 Aug 12;17:1918797. doi: 10.3389/fpsyt.2026.1918797 (PMC13506722; doi:10.3389/fpsyt.2026.1918797)
Supplement: Supplementary file 1 [file Table1.docx]

**Supplementary Table S1. Coding of Variables Included in the Regression Models**

| **Variable** | **Operational definition** | **Coding/scale** | **Reference category or interpretation** |
| --- | --- | --- | --- |
| Mean standardized symptom level | Average of the maternal and paternal standardized subscale scores for the corresponding symptom domain. | Continuous: (maternal z-score + paternal z-score) / 2. | Higher values indicate a higher mean standardized symptom level; no reference category. |
| Body weight | Child body weight recorded at baseline assessment. | Continuous, kilograms. | Higher values indicate greater body weight; no reference category. |
| Foster care history | Whether the child had a history of foster care before the baseline assessment. | Binary: 0 = no; 1 = yes. | Reference: no foster care history. |
| Delivery mode | Mode of delivery at birth. | Binary: 0 = vaginal delivery; 1 = cesarean delivery. | Reference: vaginal delivery. |
| Paternal childhood ADHD-like symptoms | Father's retrospective report of having ADHD-like symptoms during his own childhood; this was not a clinically confirmed ADHD diagnosis. | Binary: 0 = no; 1 = yes. | Reference: no retrospectively reported childhood ADHD-like symptoms. |
| Maternal childhood ADHD-like symptoms | Mother's retrospective report of having ADHD-like symptoms during her own childhood; this was not a clinically confirmed ADHD diagnosis. | Binary: 0 = no; 1 = yes. | Reference: no retrospectively reported childhood ADHD-like symptoms. |
| Sleep problems | Parent-reported presence of child sleep problems at baseline. | Binary: 0 = no; 1 = yes. | Reference: no sleep problems. |
| Biological family status | Whether the child was living in the biological family at baseline. This variable does not indicate whether questionnaire respondents were biological parents. | Binary: 0 = biological family; 1 = non-biological family. | Reference: biological family. |
| Paternal education | Father's highest reported educational level. | Ordinal: 1 = high school or below; 2 = college or bachelor's degree; 3 = postgraduate degree or above. | Treated as an ordinal predictor; no reference category. |
| Maternal familiarity with the child | Mother's self-rated familiarity with the child, assessed using a single questionnaire item. | Ordinal: 1 = unfamiliar; 2 = relatively familiar; 3 = familiar. | Treated as an ordinal predictor; higher scores indicate greater familiarity. |
| Child sex | Sex of the child recorded at baseline. | Binary: 0 = female; 1 = male. | Reference: female. |
| Birth injury | Parent-reported history of birth injury or birth trauma. | Binary: 0 = no; 1 = yes. | Reference: no birth injury. |
| Maternal education | Mother's highest reported educational level. | Ordinal: 1 = high school or below; 2 = college or bachelor's degree; 3 = postgraduate degree or above. | Treated as an ordinal predictor; no reference category. |
| Somatic illness after the neonatal period | Parent-reported history of somatic illness in the child after the first month of life. | Binary: 0 = no; 1 = yes. | Reference: no reported somatic illness after the neonatal period. |
| Full-term birth | Whether the child was born at full term. | Binary: 0 = preterm; 1 = full-term. | Reference: preterm birth. |
| Maternal severe physical illness | Questionnaire-reported history of severe physical illness in the mother; the report was not independently verified using medical records. | Binary: 0 = no; 1 = yes. | Reference: no reported maternal severe physical illness. |
| Maternal belief that the child had ADHD | Mother's belief that the child had ADHD, assessed with a single item during the baseline clinical assessment before diagnostic feedback. | Ordinal: 1 = definitely not; 2 = possibly; 3 = definitely. | Higher values indicate a stronger maternal belief that the child had ADHD. |
| Paternal belief that the child had ADHD | Father's belief that the child had ADHD, assessed with a single item during the baseline clinical assessment before diagnostic feedback. | Ordinal: 1 = definitely not; 2 = possibly; 3 = definitely. | Higher values indicate a stronger paternal belief that the child had ADHD. |
| Maternal perceived parenting consensus | Mother's perception of whether she and the father could reach consensus in child-rearing, assessed with a single item. | Ordinal: 1 = not at all; 2 = not very much; 3 = mostly; 4 = completely. | Higher values indicate greater maternally perceived parenting consensus. |
| Paternal perceived parenting consensus | Father's perception of whether he and the mother could reach consensus in child-rearing, assessed with a single item. | Ordinal: 1 = not at all; 2 = not very much; 3 = mostly; 4 = completely. | Higher values indicate greater paternally perceived parenting consensus. |

*Note.* ADHD, attention-deficit/hyperactivity disorder. Binary indicators were entered so that the stated reference category represented the baseline comparison group. Ordinal predictors were entered using their ordered questionnaire codes, with higher values indicating higher levels of the corresponding construct. Parental childhood ADHD-like symptoms were based on retrospective self-report rather than confirmed diagnoses. Maternal severe physical illness was based on questionnaire report rather than independent medical verification.
