# Supplementary material for: Direction and magnitude of mother–father discrepancies in ADHD symptom ratings: associated factors in a child and adolescent mental health sample
Source: Front Psychiatry. 2026 Aug 12;17:1918797. doi: 10.3389/fpsyt.2026.1918797 (PMC13506722; doi:10.3389/fpsyt.2026.1918797)
Supplement: Supplementary file 2 [file Table2.docx]

**Supplementary Table S2. Sensitivity Analyses Using Raw Signed Mother–Father Discrepancy Scores Across IA, HI, and OD Symptoms**

| **Associated factors** | **IA: B (95% CI); β; p** | **HI: B (95% CI); β; p** | **OD symptoms: B (95% CI); β; p** |
| --- | --- | --- | --- |
| **Demographic and clinical factors** | | | |
| Mean standardized symptom level | 0.110 (−0.622, 0.842); 0.015; .767 | 0.283 (−0.328, 0.894); 0.045; .363 | −0.172 (−0.682, 0.338); −0.032; .508 |
| Body weight | −0.029 (−0.074, 0.016); −0.059; .205 | — | — |
| Foster care history | 1.390 (−0.093, 2.873); 0.087; .066 | — | 1.308 (0.226, 2.390); 0.109; .018 |
| Delivery mode | −0.329 (−1.495, 0.837); −0.026; .580 | — | — |
| Paternal childhood ADHD-like symptoms | −1.218 (−2.569, 0.134); −0.082; .077 | −1.036 (−2.232, 0.161); −0.080; .090 | — |
| Maternal childhood ADHD-like symptoms | — | −0.890 (−2.286, 0.506); −0.059; .211 | — |
| Sleep problems | 0.630 (−0.530, 1.790); 0.050; .287 | — | 0.826 (−0.045, 1.697); 0.087; .063 |
| Biological family status | 0.887 (−1.400, 3.174); 0.035; .446 | — | — |
| Paternal education | 0.514 (−0.497, 1.524); 0.046; .318 | 0.328 (−0.705, 1.361); 0.034; .533 | — |
| Maternal familiarity with the child | −1.929 (−4.123, 0.264); −0.081; .085 | — | — |
| Child sex | — | 0.900 (−0.362, 2.163); 0.066; .162 | 1.317 (0.276, 2.359); 0.112; .013 |
| Birth injury | — | −0.782 (−2.853, 1.289); −0.035; .458 | — |
| Maternal education | — | 0.513 (−0.581, 1.608); 0.051; .357 | 0.372 (−0.410, 1.153); 0.043; .351 |
| Somatic illness after the neonatal period | — | — | 0.687 (−0.141, 1.515); 0.074; .104 |
| Full-term birth | — | — | 1.115 (−0.293, 2.522); 0.071; .120 |
| Maternal severe physical illness | — | — | 1.835 (−1.155, 4.826); 0.055; .228 |
| **Parental appraisal and family-context variables** | | | |
| Maternal belief that the child had ADHD | 1.269 (0.194, 2.344); 0.121; .021 | 0.142 (−0.785, 1.069); 0.016; .763 | 1.122 (0.352, 1.891); 0.143; .004 |
| Paternal belief that the child had ADHD | −1.906 (−2.887, −0.925); −0.193; <.001 | −1.134 (−2.003, −0.266); −0.133; .011 | −0.798 (−1.530, −0.065); −0.108; .033 |
| Maternal perceived parenting consensus | −0.722 (−1.524, 0.081); −0.086; .078 | −0.775 (−1.464, −0.087); −0.107; .027 | −0.919 (−1.510, −0.327); −0.147; .002 |
| Paternal perceived parenting consensus | 1.288 (0.307, 2.270); 0.124; .010 | 0.722 (−0.142, 1.586); 0.080; .101 | 0.974 (0.248, 1.701); 0.125; .009 |

*Note.* B denotes the unstandardized regression coefficient in raw-score discrepancy units; CI, confidence interval; β, standardized regression coefficient; IA, inattention; HI, hyperactivity/impulsivity; OD symptoms, oppositional defiant symptoms. Raw signed discrepancy scores were calculated as maternal raw scores minus paternal raw scores; therefore, positive coefficients indicate a more positive mother–father difference (relatively higher maternal ratings), whereas negative coefficients indicate a more negative difference (relatively higher paternal ratings). Mean standardized symptom level was calculated as the average of the maternal and paternal z-scores for the corresponding domain. The same predictor sets used in the primary standardized signed discrepancy models were retained. Model fit was as follows: IA, F(13, 446) = 3.303, p < .001, R² = .088, adjusted R² = .061; HI, F(11, 447) = 2.397, p = .007, R² = .056, adjusted R² = .032; OD symptoms, F(12, 446) = 4.448, p < .001, R² = .107, adjusted R² = .083. Dashes indicate that the variable was not included in the final multivariable model for that symptom domain. Model-specific sample sizes were 460 for IA and 459 for HI and OD symptoms because of missing covariate data. Higher scores on ordinal variables indicated higher levels of the corresponding construct. Detailed coding is provided in Supplementary Table S1.
